# Effects of Moving the Irradiation Isocenter From the Lesion to the Center of the Brain on High-Definition Dynamic Radiosurgery Planning With Volumetric-Modulated Arcs for Single Brain Metastases

**DOI:** 10.7759/cureus.89380

**Published:** 2025-08-04

**Authors:** Kazuhiro Ohtakara, Kojiro Suzuki

**Affiliations:** 1 Department of Radiation Oncology, Kainan Hospital Aichi Prefectural Welfare Federation of Agricultural Cooperatives, Yatomi, JPN; 2 Department of Radiology, Aichi Medical University, Nagakute, JPN

**Keywords:** brain metastases, dose conformity, dose gradient, dose inhomogeneity, high-definition dynamic radiosurgery, isocenter position, monitor units (mu), radiotherapy treatment planning, stereotactic radiosurgery, volumetric-modulated arc therapy

## Abstract

Background

This study was conducted to examine the effects of moving the isocenter (IC) position from the lesion to the center of the brain on stereotactic radiosurgery (SRS) planning with volumetric-modulated arcs (VMA) using the High-Definition Dynamic Radiosurgery (HDRS) platform, a combination of the Agility^®^ multileaf collimator (MLC) (Elekta AB, Stockholm, Sweden) and the Monaco^®^ planning system (Elekta AB), for single brain metastases (BMs).

Methodology

The study subject included 36 clinical BMs with the gross tumor volume (GTV) ranging from 0.04 to 48.09 cc (median 7.91 cc). Two VMA plans were prepared for each GTV under identical conditions except for the location of the IC as follows: the center of each GTV (IC_GTV) vs. the center of the brain (IC_Brain). The same prescription dose was uniformly assigned to each GTV *D*_V-0.01 cc_, the minimum dose to a GTV minus 0.01 cc (*D*_>95%_), for GTV >0.20 cc and *D*_95%_ for GTV ≤0.20 cc.

Results

The GTV values on the dose-volume histograms (DVHs) were different from the same contoured volumes, and the differences were considerably different between the IC_GTV and the IC_Brain, ranging from -0.07 to 0.14 cc. The inter-IC distances significantly decreased as the depths of the GTV increased. The total calculation times (tCT) and total monitor units (MU) per fraction were significantly longer and higher in the IC_Brain than in the IC_GTV, respectively. The MU differences significantly increased as the IC distances increased. The dose 2 mm inside the GTV boundary was significantly higher in the IC_Brain than the IC_GTV, while there were no significant differences in the other metrics relevant to the dose conformity and gradients outside and inside the GTV boundary. The lack of significant differences in the dose distributions was mainly attributed to some of the leaf widths of essentially <5 mm, variable by the dynamic involvement of diaphragms in the segment formation, and the different combinations of MLC angles with 0º, 45º, and 90º.

Conclusions

HDRS with VMA created similar dose distributions for SRS of single BMs, irrespective of the IC position, except for the steepness of dose increase just inside the GTV boundary. However, moving the IC from the lesion to the center of the brain significantly increased the tCT and total MU per fraction and was associated with a substantial change in the GTV value ​​on the DVH. Positioning the IC to the center of each lesion is generally recommended for VMA-based HDRS of single BMs.

## Introduction

Multiple brain metastases (BMs) may occur in cancer patients synchronously with initial diagnosis, metachronously after the initiation of treatment, or after prophylactic or therapeutic whole-brain irradiation (WBI) [1,2]. WBI ≥30 Gy has still generally been used for patients harboring five or more multiple radiation-naïve BMs [1,3]. After WBI, neurocognitive decline accompanied by morphological changes such as brain parenchymal atrophy and white matter degeneration is more likely to become apparent over time, in addition to the inevitable acute toxicity, even if there is no recurrence or new manifestation of BMs [3,4]. In addition, the antitumor effect of WBI ≥30 Gy is frequently transient and limited unless sensitization by systemic therapy is achieved. The ultimate goal of treating BMs is to preserve neurocognitive function through treatment of existing disease and prevention of new lesions [4]. Therefore, the trend towards treating multiple BMs primarily with single- or multi-fraction stereotactic radiosurgery (SRS) in seamless coordination with systemic therapy is gaining momentum, in the hope of controlling small or microscopic BMs with systemic therapy [5-9].

In SRS using a general-purpose linac, there has been remarkable progress in the technique of simultaneous irradiation using dynamic conformal arcs (DCA) or volumetric-modulated arcs (VMA) with a single isocenter (IC) for multiple BMs [2,10-16]. In particular, VMA is an essential technique for maximizing the quality and efficiency of treatment planning for SRS using a 5-mm leaf-width multileaf collimator (MLC) [10,17]. The High-definition Dynamic Radiosurgery (HDRS) platform, with a combination of the Agility^®^ MLC (Elekta AB, Stockholm, Sweden) and the Monaco^®^ planning system (Elekta AB), enables simple and efficient implementation of the single-IC multi-target SRS with all 5-mm leaf-width wide irradiation fields and treatment plan templating [10,17,18]. In single-IC multitarget SRS, most of the lesion centers are located at different positions with various distances from the IC, which inevitably leads to differences in the pattern of leaf adaptation to each target boundary and segment formation [10,12]. Furthermore, the differences may vary depending on the characteristics of the device and planning system used, and experience and skills of the treatment planners [19,20].

This planning study was therefore conducted to examine the effects of moving the IC position from the center of the lesion to the center of the brain on VMA-based HDRS planning for single BMs. Specifically, the differences in the quality of dose distributions and the efficiency of the planning and delivery were compared.

## Materials and methods

This study was approved by the Clinical Research Review Board of Kainan Hospital, Aichi Prefectural Welfare Federation of Agricultural Cooperatives (approval number: 20240830-01).

The study subjects were 36 lesions in 28 patients extracted from cases previously treated at our hospital, and some of them overlapped with those in previous studies. Each lesion was treated as a single BM. Each gross tumor volume (GTV) was delineated using the MIM Maestro^®^ version 7.1.3 (MIM Software, Inc., Cleveland, OH, USA), based on the T2/postcontrast T1 matching [18]. The GTV ranged from 0.04 cc to 48.09 cc (median value: 7.91 cc; interquartile range [IQR]: 2.10, 23.22 cc). The depth of the GTV was measured as the distance from the GTV center to the nearest head surface [18].

The treatment system consisted of the following: the Infinity^®^ linac (Elekta AB) with a flattening filter-free mode of a 6 MV X-ray beam and a 160-leaf 5-mm leaf-width MLC Agility^®^ (Elekta AB); and the treatment planning system was Monaco^®^ version 5.51.10 (Elekta AB) [10,21-23]. Two VMA plans were prepared and compared for each GTV under identical conditions except for the location of the IC as follows: the GTV center (the IC_GTV group) vs. the center of the brain (the IC_Brain group). The distance between the two isocenters (ICs) for each GTV was calculated as the square root of the sum of the squares of the distances between the left-right, anterior-posterior, and superior-inferior axes between each coordinate. The uniform arc configuration and the collimator angle settings consisted of the following: one coplanar arc with 360º rotation and the collimator angle of 0º; and two non-coplanar arcs with 180º rotation and the collimator angles of 45º and 90º [18]. The three arcs were arranged at 60º intervals to divide the cranial hemisphere into thirds [18]. The increment (Inc) parameter was set to 20º for each arc [18].

The details of the unified optimization method for VMA are shown in Table 1.

**Table 1:** Optimization settings for volumetric-modulated arcs planning using the Monaco system. The minimum volume is the coverage value of *D*_V-0.01 cc_, the minimum dose to a gross tumor volume (GTV) minus 0.01 cc (*D*_>95%_ for GTV >0.20 cc, *D*_95%_ for GTV ≤0.20 cc), for each GTV. Rx, prescription; IMRT, intensity-modulated radiotherapy; RMS, root mean square; CT, computed tomography; Max, maximum; Min, minimum

Item	Setting details		
Prescription	Rx dose (Gy): 43.000		
	Number of fractions: 5		
IMRT constraints (Pareto)	Structure	Cost function	Parameter settings
	GTV	Target Penalty	Prescription (Gy): 43.000
			Minimum volume (%): 95.00-99.98*
	Patient (body contour)	Conformality	Relative isoconstraint: 0.01
			Margin around target: 8 cm
			Multicriterial +
		Quadratic overdose	Maximum dose (Gy): 43.000
			RMS dose excess (Gy): 0.020
			Multicriterial +
			Shrink structures (cm): GTV 0.20
IMRT prescription parameters	Minimum CT number: -200		
	Auto flash margin (cm): 0.20		
	Surface margin (cm): 0.30		
	Beamlet width (cm): 0.30		
	Target margin: Normal (8 mm)		
	Avoidance margin: Normal (8 mm)		
Sequencing parameters	Segment shape optimization: +		
	High-precision leaf positions: 20		
	Max number of arcs: 1		
	Max number of control points per arc: 1,024		
	Min segment width (cm): 0.50		
	Fluence smoothing: Medium		
Calculation properties	Grid spacing (cm): 0.10		
	Calculation dose deposition to: Medium		
	Statistical uncertainty (%): 1.00 per calculation		

The optimization method was adopted based on previous studies to maximize dose conformity and dose gradients outside and inside the GTV boundary [18]. The same prescription dose of 43.000 Gy in 5 fractions was assigned to each GTV *D*_V-0.01 cc_, the minimum dose to the GTV minus 0.01 cc (*D*_>95%_) for GTV >0.20 cc or *D*_95%_ for GTV ≤0.20 cc [24]. The rescaling ratio was recorded as the change of the GTV dose to normalize the GTV coverage with the prescription dose [18]. The total calculation time (tCT) was recorded from the optimization console on Monaco^®^ [18].

The metrics for comparing dose distributions are summarized in Table 2.

**Table 2:** Evaluation metrics for comparisons of dose distributions. *This index can also be interpreted as follows: the higher the value, the steeper the dose increase inside the GTV boundary, although high values ​​may be attributed to the GTV over-coverage by a prescription isodose. *D*_V-0.01 cc_, minimum dose to a target volume (TV) minus 0.01 cc (*D*_>95%_ for TV >0.20 cc, *D*_95%_ for TV ≤0.20 cc); IDS, isodose surface *D*_0.01 cc_, minimum dose to 0.01 cc, receiving a near maximum dose, of TV (*D*_<5%_ for TV >0.20 cc, *D*_5%_ for TV ≤0.20 cc); PIV, prescription isodose volume; IIDV, irradiated isodose volume; *D*_eIIV_, minimum dose to the IIDV equivalent to a TV

Evaluation items	Metrics	Definitions	Interpretation
GTV dose inhomogeneity	GTV *D*_V-0.01 cc_ % IDS (%)	GTV *D*_V-0.01 cc_ (%) relative to the *D*_0.01 cc_ (100%)	The lower the value, the greater the heterogeneity
GTV dose conformity	PIV spillage (cc)	IIDV of GTV *D*_V-0.01 cc_ minus the GTV	The smaller the value, the better the conformity.
	GTV *D*_eIIV_ (%)	minimum dose (%) to IIDV equivalent to GTV, relative to the GTV *D*_V-0.01 cc_ (100%)	The closer the value is to 100%, the better the conformity.
	GTV *D*_eIIV_ coverage (%)	GTV coverage (%) by the *D*_eIIV_	The higher the value, the better the conformity.
Steepness of dose falloff outside the GTV boundary	GTV + 2 mm *D*_eIIV_ (%)	minimum dose (%) to IIDV equivalent to GTV + 2 mm, relative to the GTV *D*_V-0.01 cc_ (100%)	The lower the value, the steeper the dose decrease (gradient) outside the GTV boundary.
Concentric lamellarity of the dose gradient outside the GTV boundary	GTV + 2 mm *D*_eIIV_ coverage (%)	GTV + 2 mm coverage (%) by the *D*_eIIV_	The higher the value, the better the concentric lamellarity of the dose gradient outside the GTV boundary.
Steepness of the dose gradient outside the GTV	75% or 50% PIV spillage (cc)	IIDV of 75% or 50% of GTV *D*_V-0.01 cc_ minus the GTV	The smaller the value, the steeper the dose gradient outside the GTV boundary.
Steepness of dose increase inside the GTV boundary	GTV - 2 or 4 mm *D*_eIIV_ (%)	minimum dose (%) to IIDV equivalent to GTV - X mm, relative to the GTV *D*_V-0.01 cc_ (100%)	The higher the value, the steeper the dose increase inside the GTV boundary.
Concentric lamellarity of the dose gradient inside the GTV boundary	GTV - X mm *D*_eIIV_ coverage (%)	GTV - X mm coverage (%) by the *D*_eIIV_	The higher the value, the better the concentric lamellarity of the dose gradient inside the GTV boundary.

We have previously indicated issues and flaws in the following commonly used metrics: the near-minimum dose of *D*_98%_ [24,25]; the dose homogeneity index by the ICRU (the International Commission on Radiation Units and Measurements) Report 91 [25]; and Paddick’s conformity index and gradient index [26,27]. Therefore, many uncommon metrics more relevant to post-SRS efficacy and safety were included here based on previous studies [18,24,28,29]. The GTV - 2 mm and GTV - 4 mm were created only for GTVs of ≥0.50 cc (32 lesions) and ≥1.82 cc (28 lesions), respectively, with the minimum meaningful volumes for evaluation [29].

The statistical analyses were performed using the BellCurve for Excel^®^ (version 4.05; Social Survey Research Information Co., Ltd., Tokyo, Japan). Box-and-whisker plots (BWPs) were used to show the distributions of numerical variables. The whiskers denote the nearest values ≤1.5 times the IQR, and the cross marks beyond the lines indicate the outliers >1.5 times the IQR. The Wilcoxon signed-rank test (WSRT) was adopted to compare two numerical variables, many of which deviated from normal distribution. The Spearman’s rank correlation coefficient (SRCC) was used to examine the correlation between two numerical variables. The statistical significance was determined at p<0.05, and the degree was indicated on three levels: *P *< 0.05 (*), *P *< 0.01 (**), and *P *< 0.001 (***). Significant *P*-values were marked in blue in the figures.

## Results

**Figure 1:**
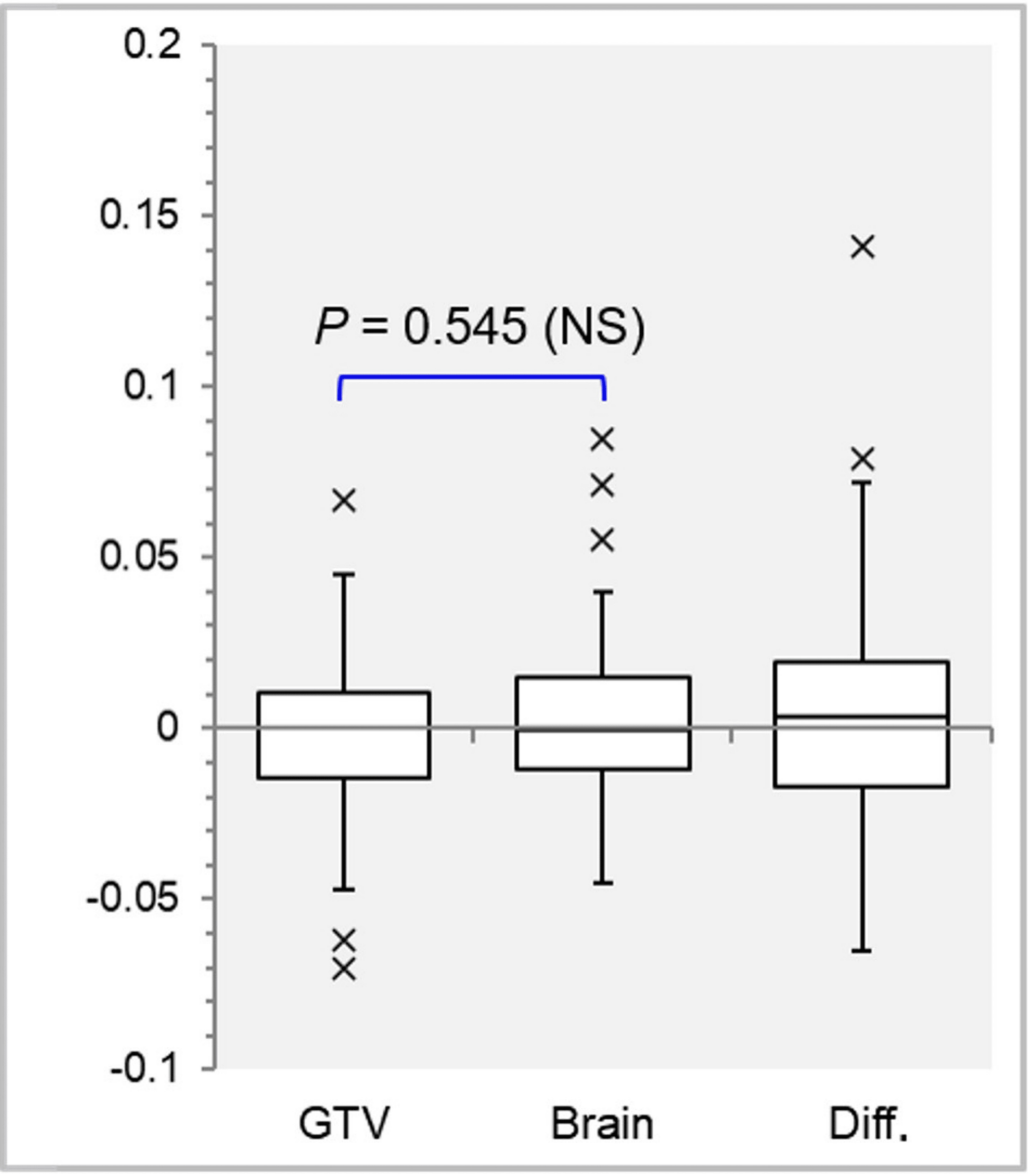
Effect of the isocenter position on gross tumor volume values between the contoured structure and the DVH. The images show box-and-whisker plots (BWPs); distributions of the gross tumor volume (GTV) differences on dose-volume histograms (DVHs) against the contoured volumes, i.e., GTV on DVH minus GTV structure, with the isocenter position being the GTV center (IC_GTV) and the brain center (IC_Brain); and the GTV differences on the DVHs (Brain minus GTV, Diff.), along with the result of the Wilcoxon signed-rank test (WSRT). diff., difference; NS, not significant

The GTV values on the dose-volume histogram (DVH) were substantially different from those of the contoured structure volumes, with the differences ranging from -0.07 to -0.05 cc to 0.07 and 0.09 cc in the IC_GTV and IC_Brain groups, respectively (Figure 1).

There was no significant difference in the GTV values between the IC_GTV and IC_Brain. There were no correlations between the GTV value differences and the GTV itself (IC_GTV: rho = -0.078, *P *= 0.650; IC_Brain: rho = 0.269, *P *= 0.112). Notably, the GTV difference between the IC_GTV and the IC_Brain (IC_Brain minus IC_GTV) considerably differed, with the volume ranging from -0.07 cc to 0.14 cc (Figure 1). In addition, there was a significant correlation between the GTV difference and the GTV (rho = 0.348, *P *= 0.038*) and between the GTV difference and the depth (rho = 0.377, *P *= 0.023*).

**Figure 2:**
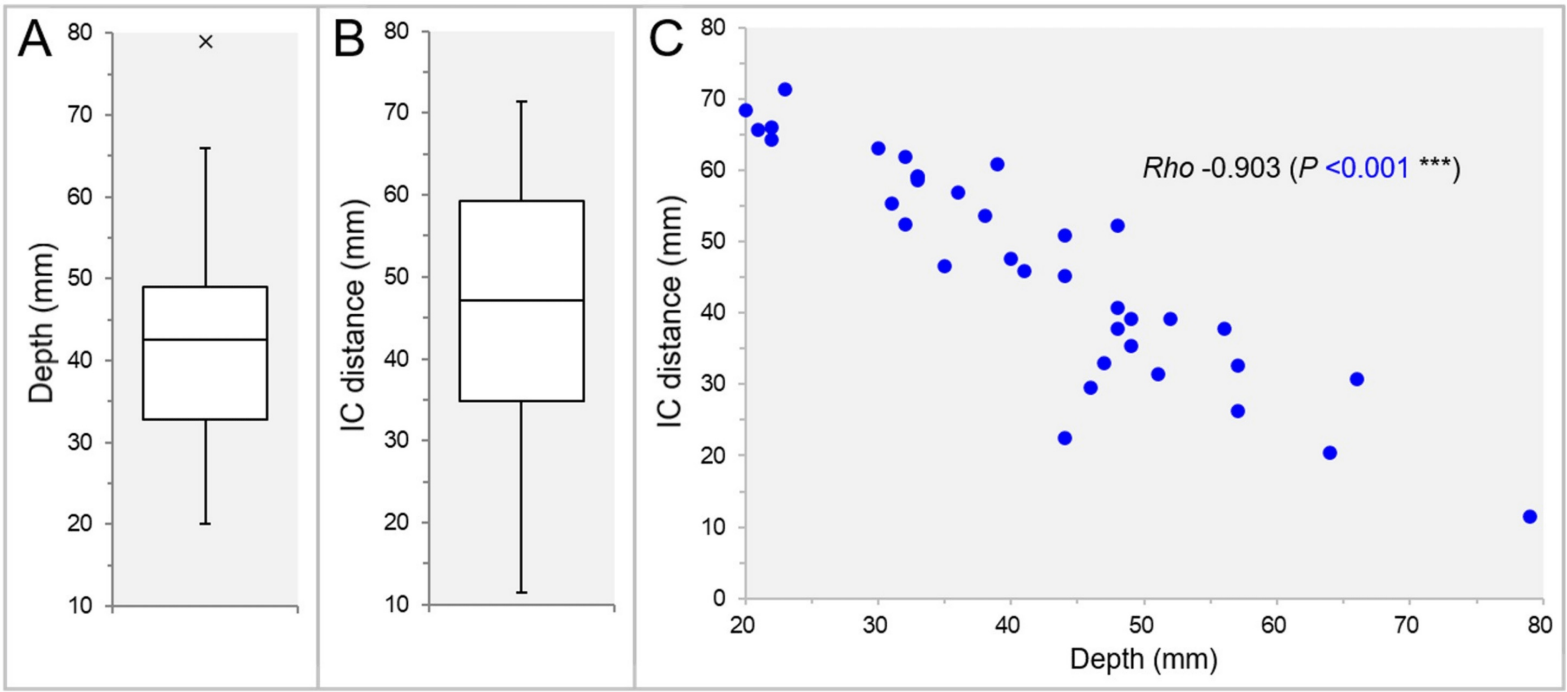
Distributions and the correlation between the lesion depths and the isocenter distances. The images show BWPs (A and B) and a scatter plot (C); distribution of the minimum depths from the head surfaces to the GTV centers (A); distribution of the isocenter (IC) distances between the IC_GTV and the IC_Brain (B); and the correlation between the GTV depths and the IC distances, along with the result of Spearman’s rank correlation coefficient (SRCC). BWP, box-and-whisker plot; GTV, gross tumor volume; IC_GTV, isocenter at GTV center; IC_Brain, isocenter at the center of the brain

The distributions of the GTV depth and the inter-IC distance are shown in Figures 2A-2B.

The IC distance decreased significantly as GTV depth increased (Figure 2C).

**Figure 3:**
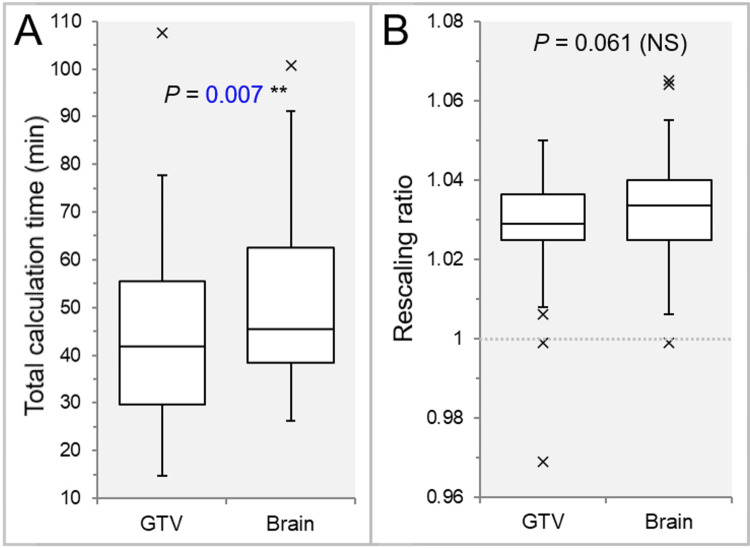
Comparison of total calculation times and rescaling ratios. The images show BWPs (A and B), along with the results of the WSRT, comparing total calculation times (A) and rescaling ratios used to normalize GTV coverage to the prescription dose (B). The dotted line in B indicates a value of 1.000, representing no need for rescaling. BWP, box-and-whisker plot; WSRT, Wilcoxon signed-rank test

The tCT was significantly longer in the IC_Brain group than in the IC_GTV group (Figure 3A), while no significant correlation was found between IC distance and the tCT difference (rho = 0.007, *P* = 0.969).

There was no significant difference in the rescaling ratio (Figure 3B), while the rescaling ratio in the GTV tended to be close to 1.000.

**Figure 4:**
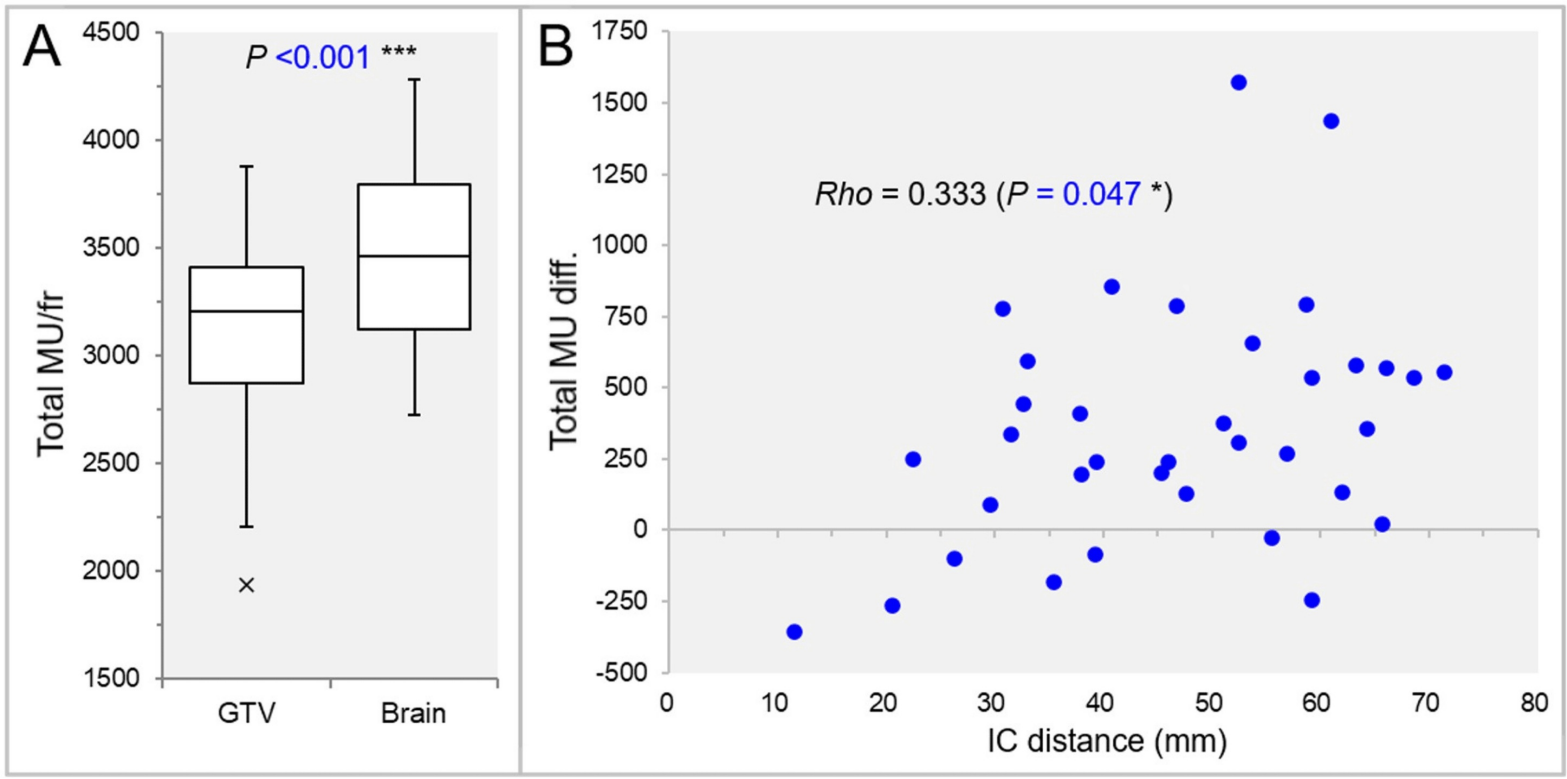
Comparisons of the total monitor units (MU) per fraction and correlation between the IC distances and the MU differences. The images show BWPs (A), along with the result of WSRT, for comparison of the total MU per fraction between the two groups (A) and a scatter plot (B), along with the result of SRCC (B), for finding the correlation between the IC distances and the total MU difference (Brain minus GTV) (B). GTV, gross tumor volume; fr, fraction; IC, isocenter; diff., difference; BWP, box-and-whisker plot; WSRT, Wilcoxon signed-rank test; SRCC, Spearman’s rank correlation coefficient

The total MU per fraction was significantly higher in the IC_Brain group than in the IC_GTV group (Figure 4A), and the MU difference increased significantly with increasing IC distance (Figure 4B).

**Figure 5:**
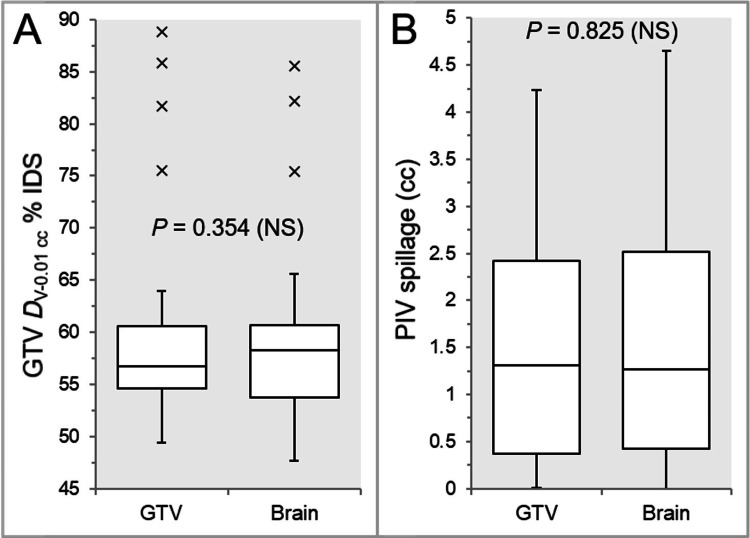
Comparison of GTV dose inhomogeneity and conformity. The images display BWPs (A and B), along with Wilcoxon signed-rank test (WSRT) results, comparing GTV *D*_V-0.01 cc_ (%)relative to the near-maximum dose (100%) (A), and the spillage of the prescription isodose volume (PIV) outside the GTV (B). GTV, gross tumor volume

There were no significant differences in the GTV dose inhomogeneity (Figure 5A) and the prescription isodose volume (PIV) spillage (Figure 5B), although the third quartile and maximum values of the PIV spillage were higher in the IC_Brain than in the IC_GTV (Figure 5B).

**Figure 6:**
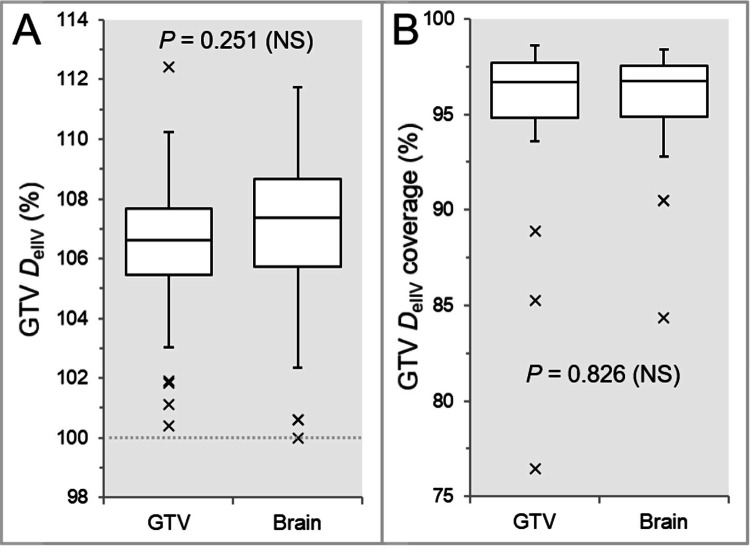
Comparison of GTV dose conformity and the steepness of dose increase just inside the prescription isodose surface. The images show BWPs (A and B), along with the results of WSRT, for comparisons of the GTV *D*_eIIV_ (%) relative to the *D*_V-0.01 cc_ (100%) (A) and the GTV coverage values by the *D*_eIIV_ (B). The dotted line in (A) shows the 100% isodose level, i.e., GTV *D*_V-0.01 cc_. GTV, gross tumor volume

There were no significant differences in the GTV *D*_eIIV_, the minimum dose to the irradiated isodose volume (IIDV) equivalent to a GTV (Figure 6A), and the GTV coverage value by the *D*_eIIV_ (Figure 6B), although the median and third quartile values of the GTV *D*_eIIV_ were higher in the IC_Brain than the IC_GTV (Figure 6A).

**Figure 7:**
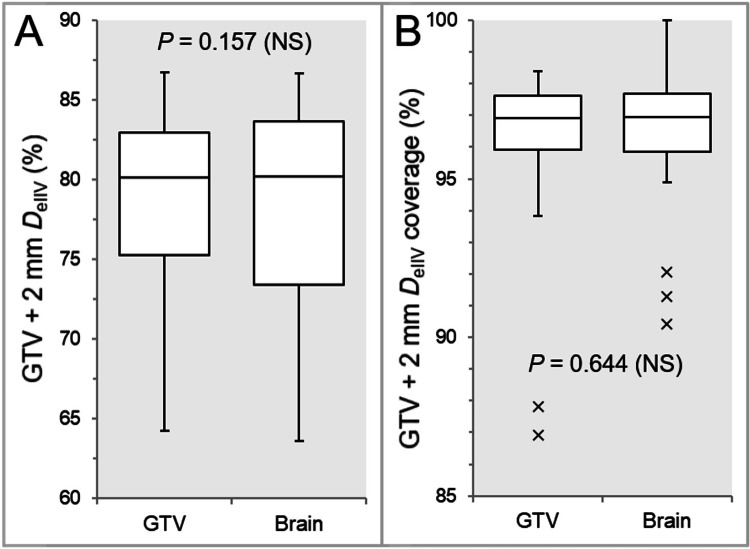
Comparisons of the appropriateness of dose attenuation margins outside the GTV boundaries. The images show BWPs (A and B), along with the results of WSRT, for comparisons of the *D*_eIIV_ of GTV + 2 mm (%) relative to the GTV *D*_V-0.01 cc_ (100%) (A) and the coverage values of GTV + 2 mm by the *D*_eIIV_ (B). GTV, gross tumor volume; BWP, box-and-whisker plot; WSRT, Wilcoxon signed-rank test

There were no significant differences in the *D*_eIIV_ of the GTV + 2 mm (Figure 7A) and the coverage value (Figure 7B).

**Figure 8:**
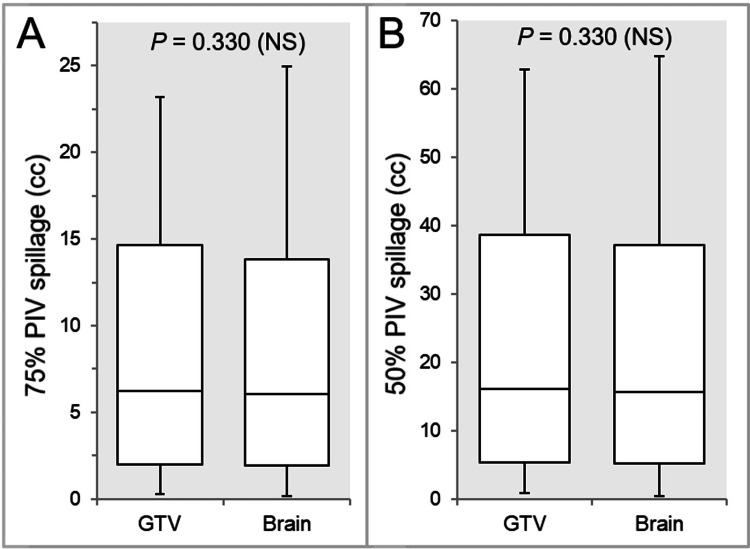
Comparisons of the steepness of dose gradients outside the GTV boundaries. The images show BWPs (A and B), along with the results of WSRT, for comparisons of the 75% (A) and 50% (B) PIV spillage volumes outside the GTV. GTV, gross tumor volume; BWP, box-and-whisker plot; WSRT, Wilcoxon signed-rank test; PIV, prescription isodose volume

There were no significant differences in the 75% (Figure 8A) and 50% (Figure 8B) PIV spillage volumes.

There were smaller trends in the IC_Brain than the GTV (20:16), while the maximum values were larger in the IC_Brain (Figures 8A-8B).

**Figure 9:**
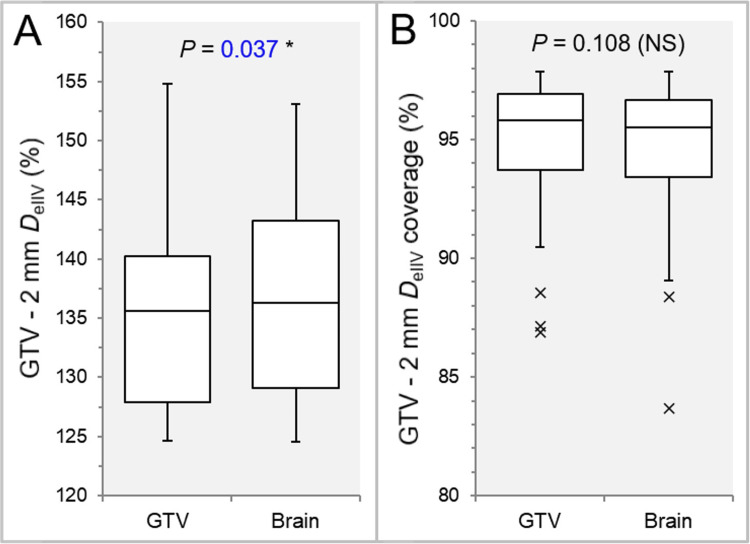
Comparisons of the steepness of dose increase and the concentric lamellarity at 2 mm inside the GTV boundaries. The images show BWPs (A, B), along with the results of WSRT, for comparisons of the *D*_eIIV_ of GTV - 2 mm (%) relative to the GTV *D*_V-0.01 cc_ (100%) (A) and the coverage values of GTV - 2 mm by the *D*_eIIV_ (B). GTV, gross tumor volume; BWP, box-and-whisker plot; WSRT, Wilcoxon signed-rank test

The *D*_eIIV_ of the GTV - 2 mm was significantly higher in the IC_Brain than the IC_GTV (Figure 9A), while there was no significant difference in the coverage value by the *D*_eIIV_, along with the higher trend in the IC_GTV (19:13) (Figure 9B).

**Figure 10:**
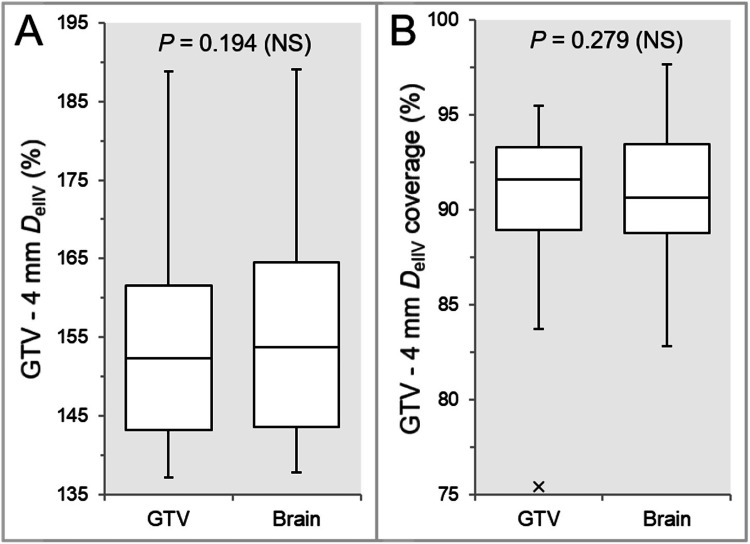
Comparisons of the steepness of dose increase and the concentric lamellarity at 4 mm inside the GTV boundaries. The images show BWPs (A, B), along with the results of WSRT, for comparisons of the *D*_eIIV_ of GTV - 4 mm (%) relative to the GTV *D*_V-0.01 cc_ (100%) (A) and the coverage values of GTV - 4 mm by the *D*_eIIV_ (B). GTV, gross tumor volume; BWP, box-and-whisker plot; WSRT, Wilcoxon signed-rank test

There were no significant differences in the *D*_eIIV_ of the GTV - 4 mm (Figure 10A) and the coverage value by the *D*_eIIV_ (Figure 10B).

The IC_Brain group had a higher trend in the GTV - 4 mm *D*_eIIV_ (17:11) and a lower trend in the coverage value (18:10).

**Figure 11:**
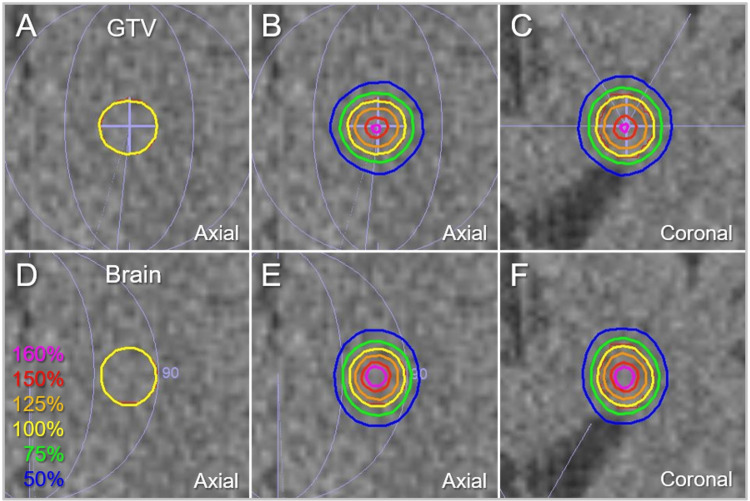
Comparison of dose distributions for a GTV of 0.72 cc. The images show computed tomographic images of the patient harboring a BM located in the paraventricular deep white matter of the left frontal lobe (A-F), onto which the barely visible GTV outline in red, arc arrangements in light purple, and representative isodoses in the IC_GTV (A-C) and the IC_Brain (D-F) groups are superimposed; axial views (A, B, D, and E); and coronal views (C and F). The isodose lines are shown as relative values to the GTV *D*_V-0.01 cc_ (*D*_98.61%_) as 100% (yellow). GTV, gross tumor volume; BM, brain metastasis

The representative dose distributions for the GTV of 0.72 cc are shown in Figure 11.

There are clear differences in the dose gradients inside and outside the GTV boundary.

**Figure 12:**
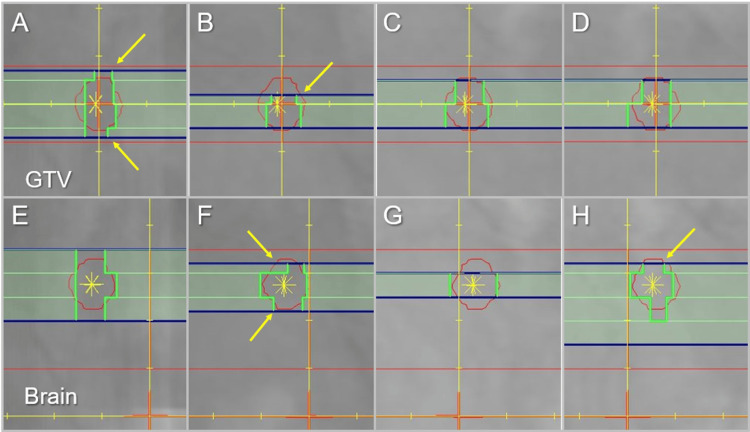
Comparison of representative beam’s eye views for a GTV of 0.72 cc. The images show the beam’s eye views (A-H) of representative four segments (light green) of coplanar arcs in the IC_GTV (A-D) and the IC_Brain (E-H) groups. Arrows (A, B, F, and H) indicate the <5 mm variable widths of some outer leaves by the dynamic involvement of diaphragms (dark blue). The asterisks show each highest dose area. GTV, gross tumor volume

The representative beam segments of the coplanar arcs are shown in Figure 12.

In the IC_GTV group, the GTV center is located between two leaf pairs, whereas in the IC_Brain group, it is positioned at the center of a single leaf pair. The outer leaves virtually function as <5 mm variable width by the dynamic involvement of diaphragms (jaws).

## Discussion

It has already been reported that the target or other structure volume value in the DVH differs from the contoured volume in the Monaco^®^ system [28,29]. This study additionally revealed that even for the same lesion, the GTV values ​​on the DVH differed when the IC position was different. Notably, the maximum difference was 0.14 cc, up to 140 voxels in a computed tomographic image with a slice thickness of 1 mm, which is equivalent to a sphere with a diameter of ≥6.4 mm. Even a difference of 0.01 cc in target volume corresponds to a sphere lesion of 3 mm in diameter, which is by no means negligible [24]. The difference in volume display due to differences in the IC position should be less than 0.01 cc, or preferably none. In other systems, it is also necessary to examine the presence and extent of variation in structural volumes on DVH evaluation. The impact of these volume differences on the objectivity of plan evaluation and the consistency of dose prescription needs to be carefully examined in the future.

In all 36 lesions, the dose distributions were visually and quantitatively different between the IC_GTV and the IC_Brain. However, there were no clinically meaningful differences in the plan evaluation, except for the steep dose gradient inside the GTV boundary in the IC_Brain. The Agility^®^ MLC has 160 leaves (80 pairs) and each leaf has a width of 5 mm at the IC [10,21]. In the Segment Shape Optimization for VMA, some of the outermost leaf pairs, perpendicular to the leaf movement, effectively function as <5 mm variable widths (Figure 12), also referred to as virtual 1 mm leaves, through the more in-depth jaw tracking [10,12]. In addition, the collimator angles of the three arcs were unified into the combination of 0º, 45º, and 90º, taking advantage of the freedom of the MLC rotation angle [18]. Thus, the three arc trajectories dividing the cephalad hemisphere of the IC into thirds with the combination of different collimator angles may essentially function as a combination of smaller beamlets equivalent to those of a 2-3 mm leaf-width micro-MLC. The lack of significant differences in the dose distributions was mainly attributed to these characteristics of HDRS with VMA. The advent of HDRS has led to a fundamental reconsideration of the conventional belief that a 2.5-mm leaf-width MLC is desirable for proper implementation of intracranial SRS.

Moving the IC position from the lesion to the center of the brain generally led to longer optimization times and increased total MU per fraction. Furthermore, the farther the lesion is from the IC, the more susceptible it becomes to the effect of rotational errors in actual irradiation [11,14]. Therefore, setting the IC to the center of the lesion is generally recommended for VMA-based HDRS of single BMs. It remains to be investigated whether shifting the IC, for example, 2.5 mm or 5 mm cranially or caudally, would result in a steeper dose gradient or other dose distribution benefits for single BMs.

Study limitations

The major limitation and future issue in this study is that various dose parameters were compared using the target volumes on the DVH, which differed depending on the position of the IC. This study only compared up to the treatment planning process, and the comparisons of actual measurement verification or irradiation times were not included [22,23,30]. It is necessary to compare the dose distribution of small radiation fields with as high accuracy as possible, considering the errors in the structure volume assessment mentioned above. Differences when using other MLCs and planning systems are also an issue for future study.

## Conclusions

HDRS with VMA created similar dose distributions for SRS of single BMs, when moving the IC position from the lesion to the center of the brain, except for the steepness of dose increase just inside the GTV boundary. Moving the IC from the lesion to the center of the brain significantly increased the tCT, total MU per fraction, and the steepness of dose increase at 2 mm inside the GTV boundary and was associated with a substantial change in the GTV value ​​on the DVH. Therefore, positioning the IC to the center of each lesion is generally recommended for VMA-based HDRS of single BMs.
